# The art of note taking with mobile devices in medical education

**DOI:** 10.1186/s12909-019-1529-7

**Published:** 2019-04-02

**Authors:** Eeva Pyörälä, Saana Mäenpää, Leo Heinonen, Daniel Folger, Teemu Masalin, Heikki Hervonen

**Affiliations:** 1 Center for University Teaching and Learning, University of Helsinki, P.O. Box 21, 00014 Helsinki, Finland; 2 Clinicum, Faculty of Medicine, University of Helsinki, P.O. Box 20, 00014 Helsinki, Finland; 3 Department of Oral and Maxillofacial Diseases, Clinicum, Faculty of Medicine, University of Helsinki, P.O. Box 41, 00014 Helsinki, Finland; 4 Faculty of Medicine, University of Helsinki, P.O. Box 63, 00014 Helsinki, Finland; 5 Department of Anatomy, Medicum, University of Helsinki, P.O. Box 63, 00014 Helsinki, Finland

**Keywords:** Note taking, Mobile learning, Digitality, Annotation, Medical and dental students

## Abstract

**Background:**

Students use mobile devices extensively in their everyday life, and the new technology is adopted in study usage. Since 2013, the University of Helsinki has given new medical and dental students iPads for study use. Simultaneously, an action research project on mobile learning started focusing on these students’ mobile device usage throughout their study years. Note taking is crucial in academic studies, but the research evidence in this area is scarce. The aims of this study were to explore medical and dental students’ self-reported study uses of mobile devices and their best practices of mobile note taking.

**Method:**

An action research project began in 2013 and followed the first student cohort (124 medical and 52 dental students) with iPads from the first until the fifth study year. We explored students’ descriptions of their most important study uses of mobile devices and their perceptions of note taking with iPads. The longitudinal data were collected with online questionnaires over the years. The answers to open-ended questions were examined using qualitative content analysis. The findings were triangulated with another question on note taking and focus-group interviews.

**Results:**

The response rates varied between 73 and 95%. Note taking was the most frequently and consistently reported study use of iPads during the study years. While taking notes, students processed the new information in an accomplished way and personalised the digital learning materials by making comments, underlining, marking images and drawing. The visual nature of their learning materials stimulated learning. Students organised the notes for retention in their personalised digital library. In the clinical studies, medical students faced the teachers’ resistance and ambivalence to mobile device usage. This hindered the full-scale benefit of the novel technology in the clinical context.

**Conclusions:**

Efficient digital note taking practices were pivotal to students in becoming mobile learners. Having all their notes and learning materials organised in their personal digital libraries enabled the students to retrieve them anywhere, anytime, both when studying for examinations and treating patients in the clinical practice. The challenges the medical students met using mobile devices in the clinical setting require further studies.

**Electronic supplementary material:**

The online version of this article (10.1186/s12909-019-1529-7) contains supplementary material, which is available to authorized users.

## Background

Students use mobile devices extensively for communication and information seeking in their everyday life. With the development of mobile technology and students’ self-directed study use of smart phones and tablet computers, several medical schools have incorporated mobile devices into their learning environment [1, 2]. Tablet computers, especially iPads, have been piloted and these devices have been reported to invigorate students’ information seeking, time-management and note taking [2–5]. Students have had mostly positive attitudes towards mobile learning [6]. They have benefited from using mobile devices as an online information resource [7, 8], valued having digital course materials always at hand and expressed that information technology in classes improved their learning [9]. Applications for mobile devices have been developed to assist students and clinicians in clinical decision making and have provided students with timely feedback in the workplace [10–12].

The art of note taking is crucial in academic studies but research on how students take notes with the digital technology is scarce [13]. While taking notes, learners interpret, filter and process the information at hand, make connections between new information and their prior knowledge and produce a format that enables them to retrieve information later. The seminal work by Di Vesta and Gray [14] showed that note taking served primarily two functions: encoding and storage. In the act of note taking, students encoded information by actively transcribing, selecting and summarizing relevant information. The second function was the organising and storing of information for later retrieval. Subsequent studies [15] showed that students’ proficient self-produced note taking practices led to efficient studying, improved retention and learning outcomes.

For centuries, the students’ task was to take notes with a blank paper and a pen with which to record as much information as they could, following the order of the instructor’s presentation. This type of linear note taking recorded all the content of the lectures in the order in which it was received. However, the paper and pen method also enabled non-linearity, i.e., moving back and forth in their notes, underlining, drawing and making visual representations and connections between different parts of the notes [16].

Over recent decades, teachers began to deliver students printed handouts of their lectures. Students underlined, made comments and complemented the condensed information the teachers provided instead of selecting, writing down and synthesizing the information themselves. Along with the digitalisation of the learning environment, teachers’ handouts were delivered in advance in an electronic format, and it was for the students to decide whether they printed the handouts or learned effective ways of using electronic annotation tools [17].

Note taking practices changed when students began to use laptops in the classroom. Mueller and Oppenheimer [18] compared longhand and laptop strategy and reported that the use of laptops led to the verbatim repetition of teachers’ speech instead of active information processing. Laptop writing advanced linearly, whereas handwriting enabled the student to make connections between sections of notes and draw. Further studies [19] claimed that the students who took notes using paper and pen performed better than those who took notes digitally. In addition, they reported that when the students were online, multitasking had a detrimental impact on learning.

The new technology and digitalised learning materials call for research on the students’ mobile note taking practices [20]. When the first generation of students using tablet computers was asked about their favoured handouts format, they declared preferring printed over digital handouts [3]. Ellaway and her colleagues [21] reported that less than a third of medical students to whom mobile devices were delivered used them for note taking. Instead, they favoured laptops and paper-based notes. Students’ ability to use mobile technology for note taking varied and students needed support for the active usage of these devices for educational purposes [21, 22].

Students’ mobile learning strategies are still evolving. It is vital for both the students and teachers to update their perceptions of efficient note taking strategies. In 2013, the Faculty of Medicine at the University of Helsinki began to deliver tablet computers to first-year medical and dental students for their personal study use. Since only iPads were compatible with interactive electronic books (Inkling books) and medical applications such as three-dimensional anatomy applications, these devices were chosen over other tablet computers. At that time, the learning materials were already provided in a digital format for the students in their first two study years.

The aims of this study were to explore students’ perceptions of the study use of mobile devices and digital note taking practices in the first cohort of tablet computer users during their studies. We sought to answer the following questions: 1) What were the students’ most important self-reported study uses of mobile devices? 2) How did the note taking practices change over the study years? 3) What were the students’ perceptions of the best practices of note taking with mobile devices?

## Methods

### Research strategy

Action research was chosen as the research strategy of this project in 2013. It is a participatory and collaborative strategy designed to explore evolving practices and solve educational challenges together with the participants [23, 24]. The research group involved teachers, students, information technology and pedagogical experts and a representative of the university library. In an iterative process, the group recognized problems by collecting and analysing versatile data and communicating the possible solutions to the teaching and learning community to improve educational practices [25].

### Setting and participants

The research data were collected at the University of Helsinki in Finland. The target group was the first student cohort to whom iPads were delivered for their study use: 124 medical and 52 dental students. The cohort under scrutiny entered the Faculty of Medicine in 2013 and in 2018 were in their fifth study year. When they began their studies, 80% of the respondents had a smartphone, 21% also had another tablet computer in addition to the iPad and 77% had a laptop. The students with another tablet computer reported only the occasional every-day use of these devices. As they started studying medicine or dentistry, they rapidly adopted iPads for study purposes.

The duration of basic medical education was six years and that of dental education five and a half years. For full authorisation, students had to undergo an additional training for primary health care. The two first study years were mainly the same for medical and dental students. They studied biomedical topics in problem-based tutorials and lectures in mixed groups of medical and dental students. In their third study year, students continued separately in their respective medical and dental units. Dental students began clinical studies in the autumn term and medical students in the spring term. Clinical studies included lectures, small-group teaching, skills lab exercises, bedside/chairside teaching and hands-on clinical teaching and learning. In Finland, both medical and dental students participated in the clinical work and treated patients under supervision.

### Data and method

The data were collected with online questionnaires including closed-ended (multiple choice and 5-point Likert scale questions) and open-ended questions on the study use of mobile devices. There were no suitable validated questionnaires available on review of the literature. The questionnaires were developed from themes arising from the literature and the teaching and learning practices in our unit. In the first and second study years, students completed the survey in autumn and in the third, fourth and fifth study years, the data were collected in the spring term to ensure students’ experiences of clinical settings. The questionnaire was originally in Finnish. The translation of the questionnaire is provided in Additional file 1. Two members of the research group (EP and TM) also conducted three focus-group interviews in the spring of 2014 with the first-study year medical and dental students, and two focus-group interviews in the spring of 2016 with the third-year medical and dental students. In 2014, three focus-groups were mixed groups of medical and dental students, the size of focus-groups varied from four to seven students and the length of the interviews from 49 to 89 min. In 2016, we made one 46-min interview with three medical students and another 48-min interview with three dental students. The focus-group guide and questions are provided in Additional file 2. These interviews were transcribed verbatim and used to test and clarify our preliminary finding based on the surveys. Therewith, we triangulated our preliminary research results to ensure the consistency of these results [26, 27].

**Table 1:**
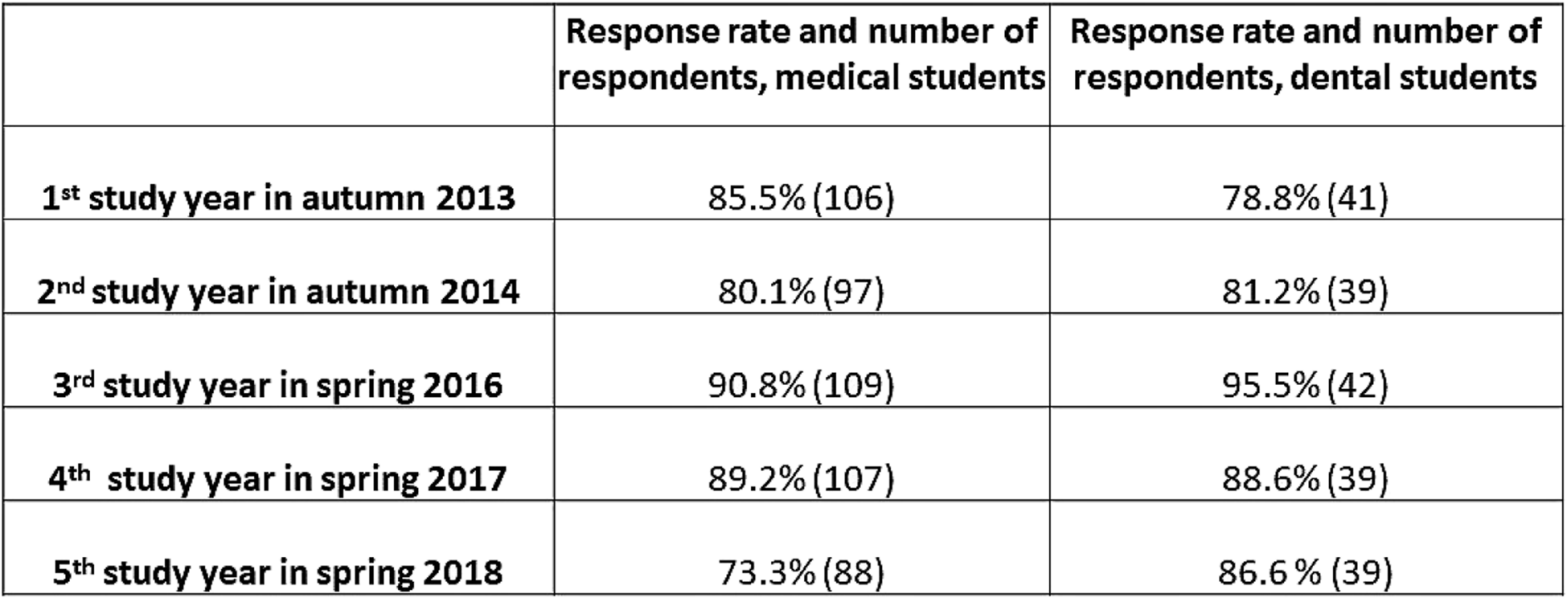
The response rates and number of participants and the response rates of the study cohort in 2013–2018

We analysed open-ended questions in which we asked the students to describe in their own words the three most important study uses of iPads. The responses were examined using inductive qualitative content analysis [28, 29]. The data consisted of more than 2000 short descriptions of students’ three most important study uses of iPads. The students’ answers were read, openly coded by two authors (EP and TM) and the first version of themes was established. Two authors (EP and TM) compared their results of coding and discussed the themes. The inter-coder agreement was high, ranging from 90 to 100%. The difference between the two coders was the lowest (90%) in coding the students’ descriptions of having notes anywhere, anytime. The authors entitled the theme “Having notes always at hand” and revised the coding in this theme. Furthermore, the two authors (EP and TM) discussed all the themes until an inter-coder agreement was reached. All themes were discussed and examined with the student authors (SM, DF and LH). To test our preliminary result of the priority of note taking in mobile learning, we analysed another item on the frequency of using the mobile device for note taking in the surveys and triangulated the findings with the transcripts of the focus-group interviews.

## Results

### The most important self-reported study uses of mobile devices

The response rates were high, ranging from 73 to 95% (Table 1). Students’ open-ended answers referring to the three most important study uses of iPads were coded and calculated in percentages to provide a comparable figure for medical and dental students. The overall percentages exceeded 100% since nearly all students named several items.

**Fig. 1:**
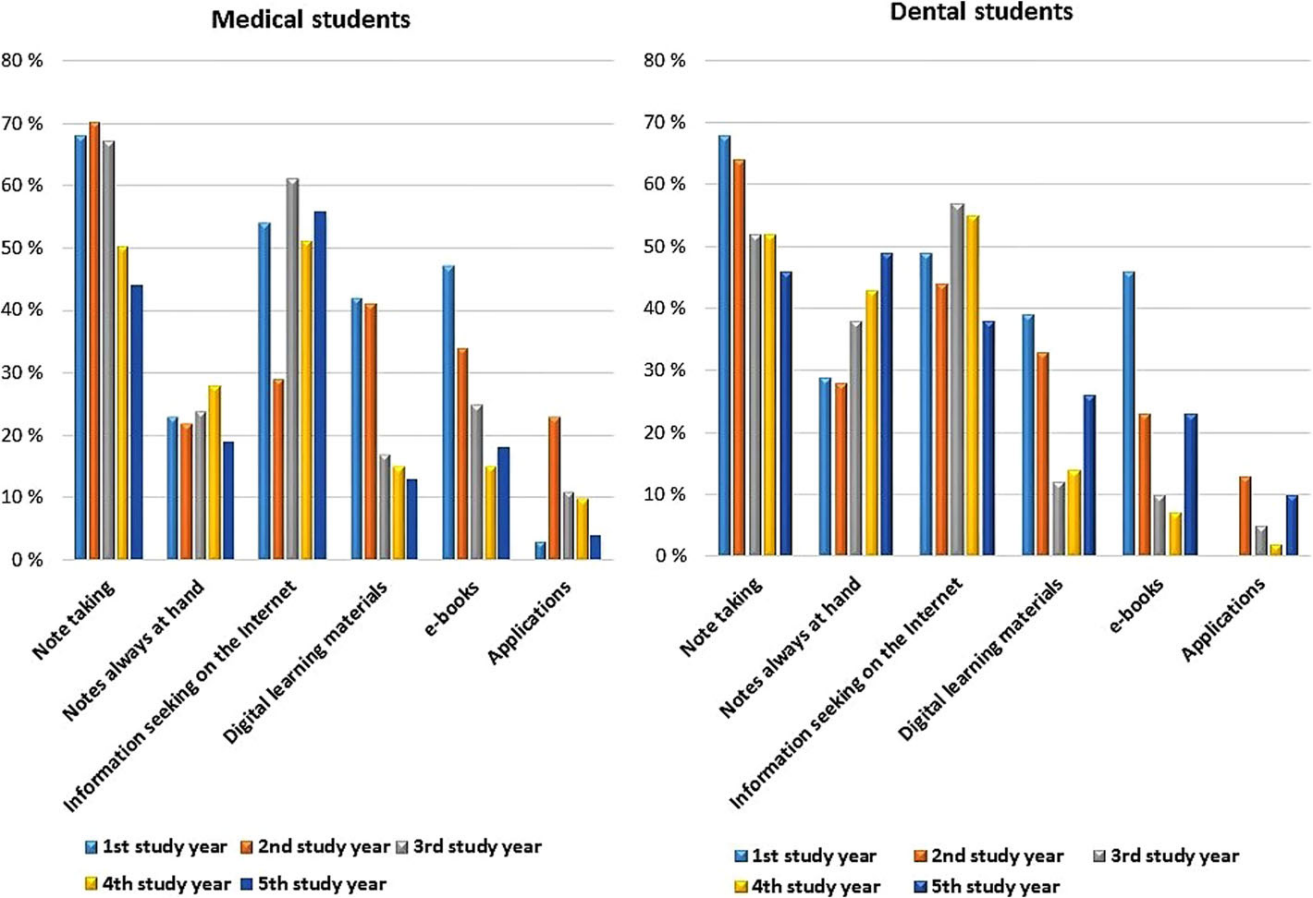
The most important self-reported study uses of mobile devices in the first student cohort using iPads (%)

In the analysis of the students’ open-ended answers about their study uses of iPads, we recognised six themes that frequently emerged in their writing and continued over the study years. These were [1] note taking, [2] notes always at hand, [3] information seeking on the Internet, [4] access to the digital learning materials, [5] use of electronic books and [6] digital applications. The two most consistently mentioned themes were note taking and information seeking on the Internet. We present the occurrence of all the six themes over the medical and dental students’ study years in Fig. 1.

Taking notes was the most frequently and consistently referred study use of iPads among the first iPad student cohort (Fig. 1). Mobile note taking was at its peak in the biomedical studies in the first and second study years. We observed a drop in the digital note taking in the clinical studies, both among the medical and dental students. The focus group interviews explained the decline in the clinical context in dentistry and medicine. In the dental clinic, note taking with the students’ own devices was forbidden in patient care for information security reasons. Otherwise, the mobile device use was well accepted. Most dental teachers revised their materials into a compatible format for iPads, produced new materials such as videos on procedures and encouraged students to use mobile devices in skills labs. Students valued having their digital notes at hand for retrieval both for preparing for the patients’ care and using the spare time between patients for studying.

In medicine, the reasons for the decline in mobile device usage given in the open-ended answers and focus group interviews were more complex than in dentistry. Medical students complained that the attitudes towards the usage of mobile devices were ambivalent; they varied from one clinical teacher to another and some teachers denied the use of mobile devices entirely. Teachers forbade using the device on the wards and students were hesitant about using them with patients. Students claimed that iPads could not be used in the wards, because they did not fit into the pockets of the white coat. When the students were allowed use the mobile devices, the learning materials were not always delivered in time or in a compatible format for annotation.

**Fig. 2:**
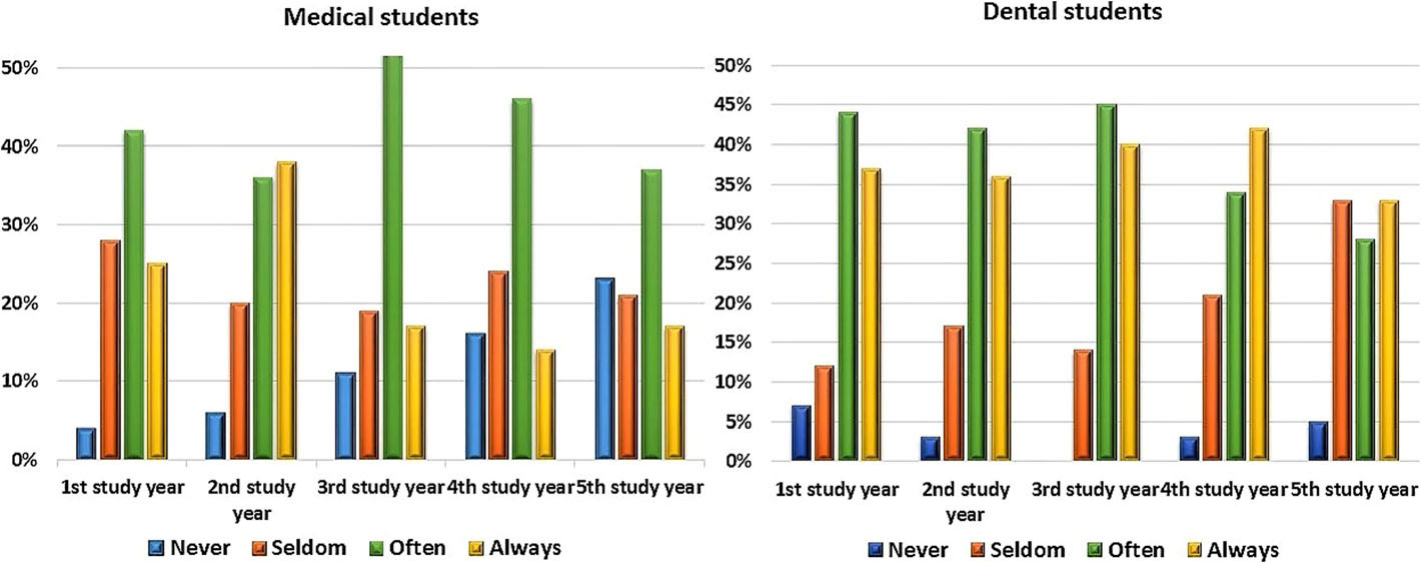
Medical and dental students’ use of iPads for note taking among the first student cohort

To test our findings based on the analysis of open-ended question and focus-group interviews, we analysed a statement in the surveys asking students to report the frequency of using the mobile device for note taking (Fig. 2). The analysis of this item confirmed that the use of the mobile device in note taking was at its highest in the second study year and that dental students used the mobile device more actively than medical students. The percentage of students who reported they always used iPads for note taking was lower among medical than dental students throughout the study years. Furthermore, the proportion of students who never used iPad for note taking increased among medical students during their study years, whereas the percentage of dental students who did not use the mobile device for note taking at all remained low during all years.

Students mostly described the note taking as processing digitalised learning materials in the classroom and retrieving notes for examinations. The students personalised the teachers’ handouts and electronic books by underlining, highlighting and commenting on them. It was vital to have all the learning materials in the mobile device compatible format before class to enable students to use the materials as a platform for their own note taking. During the first two years, all the learning materials were delivered in the digital format before class and the electronic learning environment was well designed and structured. Students appreciated the large range of digital resources. They described studying with interactive e-books, handouts and learning materials for problem-based tutorials in the digital format and using tablet computer applications such as a three-dimensional anatomy application and a note taking application (Fig. 1). They also mentioned using iPads for e-mails, problem-based learning tutorials, communication and time management, but the references to these themes were few.

Another large theme of self-reported study use of iPads was information seeking on the Internet (Fig. 1). Students valued the instant access to online information sources to consult online dictionaries or look up facts and unknown concepts. In the clinical studies, online information resources grew even more important. The students consulted databases and care guidelines for health professionals with their mobile devices, especially with their smartphones, which were always at hand. Students wished that the clinical teachers would actively recommend quality online data sources.

Students with mobile devices preferred a full-scale digital learning environment and paperlessness, did not want any printed papers or handouts nor carried textbooks to the campus, but wanted to have all the learning materials in the digital format. If students were asked to deliver a certificate of attendance, they preferred to take and send a photo instead of delivering it on paper. This pattern continued throughout their academic years. Even though mobile devices were not allowed in all clinical teaching activities, students used them for self-directed studies. Students were aware of the potential online distraction via the social media and online entertainment but reported mostly using the devices for study purposes.

### Students’ perceptions of the best practices of note taking

Students’ descriptions of their most important study usages of iPads revealed that learning to take digital notes was pivotal to medical and dental students. The crucial elements of mobile note taking were annotation and visual elements in the learning materials. Managing the learning materials was a challenge initially and the students had to develop a system to organise the notes in personal, digital libraries that could be used to retrieve information for examinations and/or clinical practice.

**Fig. 3:**
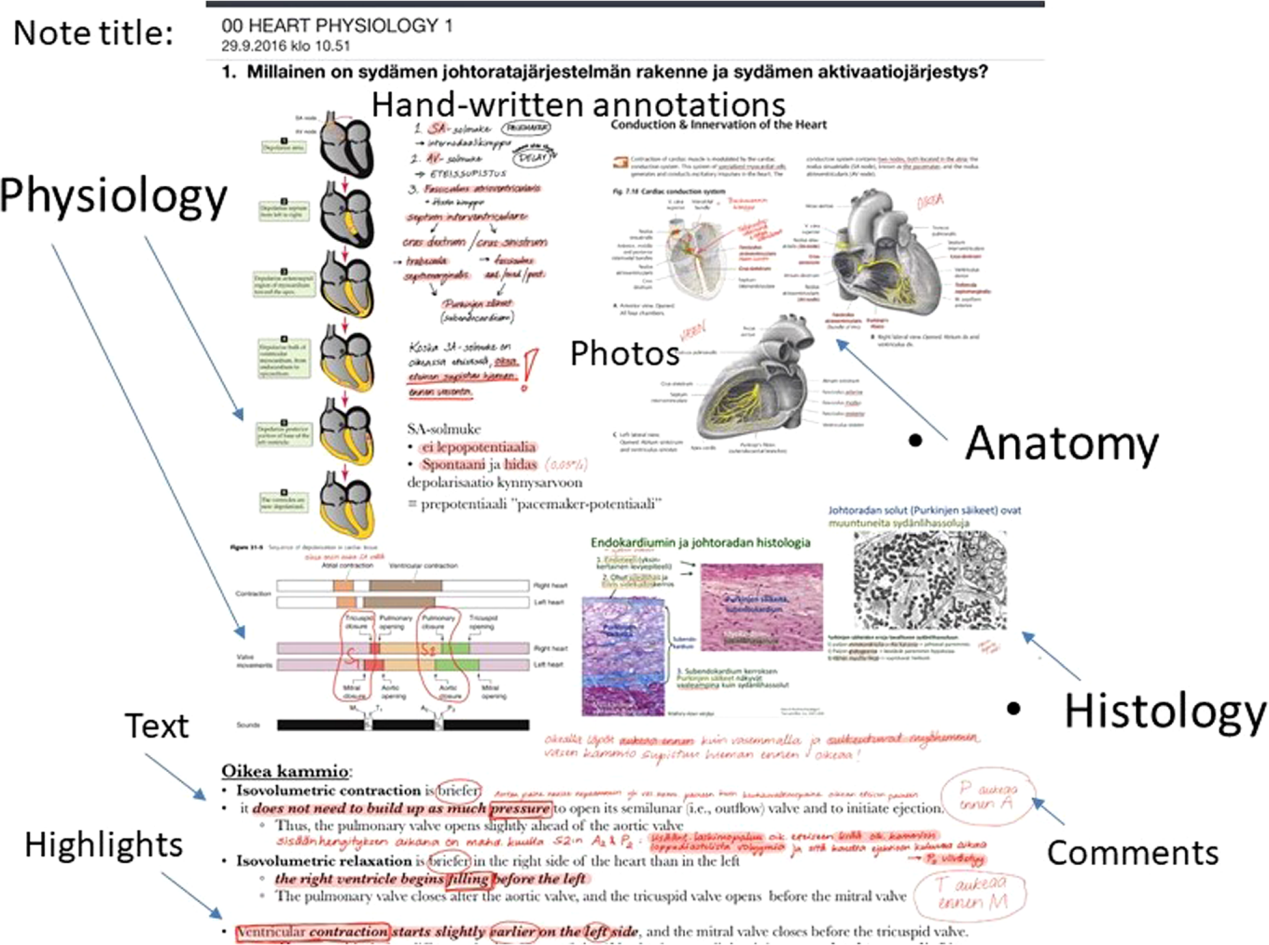
Example of student’s annotated notes

Mobile devices and digital materials profoundly transformed students’ note taking. Paper and pens were replaced by annotations with iPads using such note taking applications Notability, Evernote, OneNote, and PDF Expert. Students downloaded lecture handouts and processed the information by underlining, drawing or importing images, adding their own remarks and marginalia commentaries by typing or hand-writing with a tablet stylus or a finger (Fig. 3). Students were able to further edit the handouts by inserting photos, screen shots, charts and web links.

Traditional word-processing programmes, though used by some, offered less freedom in interaction and annotation of material and usually required the student to produce learning material from scratch instead of being able to build upon given material. The students who used laptops were often those who had previous academic studies where they had used laptops and expressed that adopting a new digital note taking strategy only caused them additional stress. Students varied their note taking strategy according to the context and access to the digital materials. They annotated the daily handouts and used a word processing application for writing minutes of meetings. Likewise, when the materials were not delivered in time or in the right format, they switched from annotation to a word processing programme.

Students valued the visual elements in handouts and other learning materials. The iPads had a fine screen resolution and provided a powerful tool for visual perception and learning by drawing. Images and photos that the students could personally annotate were crucial for learning anatomy and interpreting diagnostic images. Therefore, the students desired that the number and quality of elucidating photos and illustrations be maximised and teachers include visual assignments as parts of their learning materials by asking students to draw and complete images, describe and explain the human anatomical structures, systems and their functions. Visual assignments were successfully integrated into the second-year studies of anatomy where students collaboratively studied histological images with a virtual microscopy application.

**Fig. 4:**
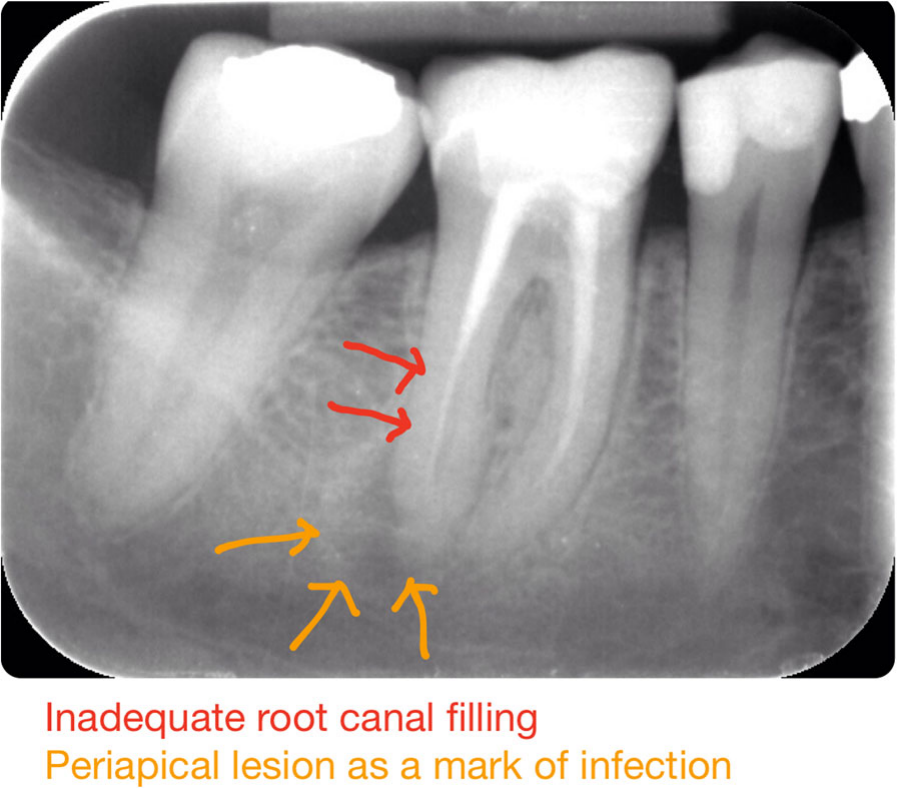
Example of student’s annotated dental X-ray

To foster their diagnostic image perception, students suggested having a medical image reservoir for self-directed studying. In Finland, dental students in their clinical studies took and interpreted X- rays, treated patients and wrote patient records accordingly. As a concrete example of the way this type of visual learning can enhance diagnostic image perception, the dental students expressed a wish for access to a reservoir of anonymous, digital dental radiographs so that they could self-directedly practise interpreting dental problems and oral infections by drawing and writing their clinical observations on the X-rays themselves (Fig. 4).

Organising notes was as important as creating them, as digital notes had to be logically arranged and easily retrievable. All note taking applications provided a personalised digital library for this purpose with search and automated backup options in cloud services (e.g., iCloud, Google Drive, DropBox). Consequently, students’ notes could be simultaneously stored in multiple locations and on different devices such as a student’s own smart phone or computer. Students created personalised digital libraries by organising their notes into thematically specific folders (e.g., Respiratory System, Cardiovascular System), and created subfolders (e.g., Anatomy, Physiology, Pharmacology) for different types of notes (handouts, written notes, articles). With progression into clinical studies, the students found that their notes became a viable tool for practical patient work, demanding categorisation more suitable for clinical practice.

Well-organised digital notes with annotations and links to additional readings and resources, made the materials more accessible and personalised. With these notes, students made connections between prior knowledge and new information; thus, their study became effective. For busy students who wanted to revise a specific topic, it sufficed to type the keyword(s) in the search function of a note taking application and display all the relevant materials. When retrieving for examinations, students valued having their notes and electronic books always at hand, during breaks between classes, seeing patients and on their way to the campus or back home.

## Discussion

As students rapidly adopt new technology in both their everyday lives and studies, medical education must seek to take advantage of its potential benefits to the learning. For five years, this research project followed the medical and dental students who began to study in the Faculty of Medicine in 2013 and were delivered iPads for their personal study use. We discovered that digital note taking was along with online information seeking the most important study uses of mobile devices. Note taking was the main usage of iPads during the first two biomedically oriented study years but dropped sharply in the clinical context, especially among the medical students.

The research evidence on mobile note taking is limited [13]. Most studies focused on handwritten notes and emphasised that self-produced notes improved retention and the learning outcomes [15]. Studies comparing handwritten and digital note taking, claimed that the students using a paper and pencil strategy outperformed digital note takers [18, 19]. More recent studies on novel technology use reported that a minority of students to whom mobile devices were delivered used them for note taking. Instead, they preferred laptops, printed handouts and paper-based notes [3, 21].

In our study, we discovered that once learned, students valued digital note taking with a mobile device. As suggested in a previous study [22], students needed support to learn to use these devices for educational purposes. At the outset of their studies, one of the questions students most frequently asked the iPad expert was related to note taking; subsequently, low threshold and tailored support was provided. We discovered that most of the students were persistent in learning to use the new device as a study tool and reservoir for learning materials.

This study revealed that mobile note taking basically served the same two functions as traditional paper and pen note taking: encoding and storage [14]. Annotation enabled students to combine the linear and non-linear note taking strategies [16]. They followed the teachers’ presentation and appreciated the potential for the non-linear way of organising the information at hand by moving back and forth in their notes, drawing, making visual representations and mind-maps. Thus, they made connections between new information and prior knowledge to foster deeper processing of ideas and strengthen the retention of content.

The longitudinal data on one student cohort provided us with a unique perspective on how the new technology was integrated into teaching and learning practices in different phases and contexts of the basic degree studies. In these years, teachers in the biomedical studies succeeded in integrating mobile devices into their teaching activities in the same way as reported in previous studies of the preclinical studies [3, 4]. In clinical study years, the compact and practice-oriented dental clinic accepted better the students’ new technology. Despite a decline in note taking in the clinical context, the dental students continued to use the mobile device in their studies.

The notable drop in medical students’ mobile device usage in the clinical studies called for attention. Medical students reported clinical teachers’ resistance and ambivalent attitudes towards the usage of iPads and mentioned that iPads did not fit into the pockets of their white coats and hence were not practical to carry in the hospitals. Students and teachers were particularly ambivalent about using the device with patients. Similar attitudes and norms discouraging the use of mobile devices at bedside have been reported in previous research [8, 10, 11] and the problems in the portability of iPads in the clinical context had also been reported earlier [22].

Positive findings of mobile device usage in the clinical context had also been reported. Mobile devices, either publicly or privately used, supported the novice doctors’ self-efficacy in the challenging clinical work [2, 11, 22, 30]. The devices were used for timely seeking online information and revising notes and electronic textbooks before encountering a patient [7]. All studies called for ethically sustainable guidelines and a transparent code of conduct for the use of mobile devices with patients.

### Trustworthiness of the study

The research data were collected using online questionnaires and were examined using inductive, qualitative content analysis [28, 29]. To improve the trustworthiness of this analysis [26, 27], two of the researchers (EP and TM) encoded the data independently, tested the inter-coder agreement and discussed the results of coding until an agreement was reached. To further test our findings, we triangulated the results of the open-ended question with the survey question concerning note taking and transcripts of focus-group interviews with medical and dental students in the first and the third study year. These three data types provided us with a fuller picture of the phenomenon studied.

We used member checking [31], involving the participants of the study to evaluate whether the results were credible from their perspective. We tested our preliminary findings of mobile note taking firstly in focus-group interviews with the students of the first study cohort in 2013 and 2016 and secondly in discussions with the student authors (SM, DF and LH). Two of them (DF and LH) were of the student cohort studied; one was a medical student and another a dental student. The student authors were indispensable in interpreting the phenomenon from the students’ perspective.

### Strengths and weaknesses of the study

The strength of our study lies in the fact that we started to collect longitudinal data from the first study cohort with mobile devices in 2013 and continued to follow their mobile learning throughout their academic years. We also constantly collaborated with both students and teachers as participants in the study; they proved to be innovative in generating new learning designs and materials for mobile devices. Thus far, we have yet to discover a comparable longitudinal and participatory study in the research literature on these subjects.

To improve the extent to which the results of research can be generalized or transferred to other contexts or settings, we provided contextual information about the project for readers to evaluate whether our findings could be transferred to their own settings [26]. We believed that our experiment of integrating iPads into student learning could be transferred to some other medical education contexts and that the results could be applicable not only to iPads but also to different kinds of mobile devices.

There were limitations in this study. One limitation was that it was performed over one student cohort at a single institution. The authors thought that this cohort was an interesting representation of the medical and dental student population but acknowledged that since this cohort was the pioneering one in new technology usage, they might have experienced more hurdles in their mobile device usage than the following ones.

### Future directions

We collected a large data set on mobile learning between 2013 and 2018. There are several questions to be analysed in the surveys and topics to be reported in our project. We will report the students’ collaboration using social media, the innovative learning design applying virtual microscopy in histology, the mobility of mobile learning, the special features of mobile learning and the hurdles in adopting mobile devices in clinical studies in medicine. This study also calls for further research on the patients’ perceptions on mobile device use in different types of healthcare settings.

## Conclusion

There were two main findings in our study. First, digital note taking was the medical and dental students’ most important study use of mobile devices during the first two study years. We mirrored our findings with the prior research on the learning functions of note taking and discovered that our students had developed refined digital note taking strategies allowing them process and personalise their learning materials and have their notes always at hand for retrieval. Second, we observed a considerable decline in the digital note taking in the clinical setting, especially among medical students. The medical students reported resistance towards the usage of iPads as they entered clinical studies*.* Identifying the hurdles to overcome in the clinical setting will enable us to discover feasible ways of using mobile devices, and to discover ways to make the most use of the potential of the new technology in the hands of the future healthcare providers.

## Additional files

Additional file 1: Message to the students and the questionnaire translated into English. (DOCX 27 kb)

Additional file 2: Focus-group interview formats translated into English. (DOCX 18 kb)
