# Supplementary material for: The art of note taking with mobile devices in medical education
Source: BMC Med Educ. 2019 Apr 2;19:96. doi: 10.1186/s12909-019-1529-7 (PMC6446288; doi:10.1186/s12909-019-1529-7)
Supplement: Supplementary file 1 — Message to the students and the questionnaire translated into English. (DOCX 27 kb) [file 12909_2019_1529_MOESM1_ESM.docx]

**Supplement 1. Message to the students and the questionnaire translated into English**

**Message to the students**

Dear medical/dental student,

The Faculty of Medicine provided you with iPads for personal study use in 2013. The Faculty established an iPad research group for developing the study use of iPads in medical and dental studies in our faculty. The iPad research group invites you to give information about the study use of iPads to improve the study use of these devices.

You are our best experts in the use of mobile devices for studying. We wish you to answer a questionnaire concerning your personal study use of the device in medical/dental studies.

Answering the questionnaire is voluntary. The answers will be treated confidentially and anonymity is guaranteed throughout the analysis and the report of the results.

You can answer the questionnaire by using the following online format. Remember to send your answers by pressing the end command at the end of the questionnaire.

<https://elomake.helsinki.fi/lomakkeet/1234/lomake.html>

We recommend you fill in the online format in a place where the wifi is stable.

Your response is valuable to us!

Eeva Pyörälä

Senior Lecturer in University Pedagogy

Faculty of Medicine

Tel. 050-3491553

e-mail: eeva.pyorala@helsinki.fi

**Questionnaire: iPads in the Faculty of Medicine, 3rd year medical/dental students**

The study use of tablet computers in medicine/dentistry is a large project started in the Faculty of Medicine in 2013 to support students’ learning. Now you are on your 3^rd^ study year and have already started your clinical studies. The aim of this questionnaire is to gather information about your personal study use of mobile devices in the clinical context and to provide us with ideas to improve the use of these devices.

By answering this questionnaire, you can help us develop the teaching and learning practices!

**Background information**

Student number ______________

The year of birth, e.g. 1990 _____________

Gender

Choose the gender

____ Female

____ Male

Programme

Choose the study programme

___ Finnish speaking

___ Swedish speaking

Do you have prior academic studies?

___ No

___ Yes

If you have prior academic studies, tell us what you have studied and how extensive your studies have been (study credits and possible degrees) ___________________________

**Students’ and teachers’ ability to use the iPad**

Assess your own ability to use the iPad in your studies at present   Not at all

Very poor

Poor

Fair

Good

Excellent

Assess the clinical teachers’ ability to use the iPad in teaching   Not at all

Very poor

Poor

Fair

Good

**Mobile devices and digitality at the clinical stage of studies**

***Learning materials***

Teachers upload lecture handouts and other learning materials before class

in the digital learning environment   Always

Often

Seldom

Never

Teachers upload the materials in the Pdf format   Always

Often

Seldom

Never

Teachers use tests for pre-assignments Always

Often

Seldom

Never

Teachers use videos for pre-assignments  Always

Often

Seldom

Never

The pre-assignments teachers use are mobile device compatible  Always

Often

Seldom

Never

Teachers tell the students about quality mobile device applications in their clinical field Always

Often

Seldom

Never

How could the use of digital learning materials be developed in clinical teaching?

______________________________

***Studies and instruction***

In class, I use mobile devices for taking notes   Always

Often

Seldom

Never

In class, I use mobile devices for seeking information Always

Often

Seldom

Never

In the skills lab, I use mobile devices for retrieving learning materials and note taking Always

Often

Seldom

Never

In class, teachers use triggers for learning, such as voting, tests and tasks Always

Often

Seldom

Never

How could the use of iPads be developed in clinical teaching?

________________________________

***Mobile devices in encounters with the patients***

I use the mobile device with patients in a way that enhances communication

with them    Always

Often

Seldom

Never

I use the mobile device to support clinical reasoning  Always

Often

Seldom

Never

I use the mobile device to support patient safety   Always

Often

Seldom

Never

I take notes of patient records with a mobile device  Always

Often

Seldom

Never

I delete the patient records from my mobile device  Always

Often

Seldom

Never

I save patient records into the cloud  Always

Often

Seldom

Never

When I use a mobile device, I take into account the risk of infection Always

Often

Seldom

Never

How could the use of iPads be developed in bedside/chairside teaching with patients?

_____________________________

***Assessment***

Teachers offer study module entrance exams suitable for mobile devices Always

Often

Seldom

Never

Teachers offer tests during the study module suitable for mobile devices  Always

Often

Seldom

Never

Teachers offer course exams online   Always

Often

Seldom

Never

How could iPads be better used in assessment for and of learning?

______________________________

**Use of social media in studies**

I use social media for delivering information  Always

Often

Seldom

Never

I use social media for arranging affairs pertaining to studies Always

Often

Seldom

Never

I use social media for sharing learning materials and resources Always

Often

Seldom

Never

I use social media for groupwork Always

Often

Seldom

Never

Describe in your own words, how the use of the social media supports your studies

__________________________

 Would you like teachers to be members of the students’ social media groups?

____ Yes

____ No

How could social media better be used in clinical studies?

_________________________

**Study applications and medical applications**

What study applications (for example note taking, sharing) do you use?

__________________________

What medical applications do you use?

__________________________

What medical applications have your teachers recommended you?

__________________________

**Studying and learning**

I print learning materials

____ a lot

____ sometimes

____ not at all

I prefer reading course literature

____ as an e-book

____ as a printed book

____ I don’t read course literature

When I study, I use at the same time (you can choose several options)

___ a printed book

___ e-book

___ my own notes

___ somebody else’s notes

___ iPad

___ Internet

___ lecture materials

___ dictionary

___ other

If you answered other, what do you refer to? __________________

When I study for an exam, I use at the same time (you can choose several options)

___ my own notes

___ somebody else’s notes

___ summaries made by the teacher

___ publicly available summaries

___ a text book

___ other

If you answered other, what do you refer to? __________________

When I study for an exam, I prefer studying

___ alone

___ with my close peers

___ with my teaching group

___ otherwise

If you answered otherwise, what do you refer to? __________________

Consider your studying and learning and answer the following statements

I believe I will do well in my studies  Fully disagree

Somewhat disagree

Neither agree nor disagree

Somewhat agree

Fully agree

I am certain I can understand the most difficult material in my studies Fully disagree

Somewhat disagree

Neither agree nor disagree

Somewhat agree

Fully agree

I am confident I can understand the basic concepts of my own field

of study  Fully disagree

Somewhat disagree

Neither agree nor disagree

Somewhat agree

Fully agree

I expect to do well in my studies  Fully disagree

Somewhat disagree

Neither agree nor disagree

Somewhat agree

Fully agree

I’m certain I can learn well the skills required in my field of study  Fully disagree

Somewhat disagree

Neither agree nor disagree

Somewhat agree

Fully agree

I believe that I will easily learn the use of the technical devices

I need at work  Fully disagree

Somewhat disagree

Neither agree nor disagree

Somewhat agree

Fully agree

Learning to use technical devices

I learn to use technical devices

____ by watching videos

____ by taking a course

____ by asking a friend

____ in another ways

If you answered in another way, tell us about it _________________________

I use iPad for studying (you may choose several options)

___ before leaving for school

___ on my way to school

___ between the classes

___ after the school day is over on campus

___ in the campus library

___ on my way home

___ at home after the school day

___ in the evening at home

___ elsewhere

If you answered elsewhere, tell us more about it _________________________

Describe your three most important study uses of iPads

1. _________________________________________________________________________
2. _________________________________________________________________________
3. _________________________________________________________________________

**Consent to participate in the study**

We hope that we can use the answers of this questionnaire in a study about the use and effectiveness of the tablet computers and are able to combine the answers with student records and consequent questionnaires of the iPad research project. Full confidentiality and anonymity in the analysis and reporting of the research material are guaranteed.

I give my consent for using the answers given in this survey in a study and collate the answers with my student records and consequent questionnaires of the iPad research project.

Consent

____ Yes

____ No

Thank you for answering the questionnaire!

Inquiries concerning the research and the questionnaire:

Eeva Pyörälä, eeva.pyorala@helsinki.fi, puh. 050-3491553

Teemu Masalin, teemu.masalin@helsinki.fi

Remember to press the ‘send’ button at the end of the webpage.
