# Supplementary material for: The art of note taking with mobile devices in medical education
Source: BMC Med Educ. 2019 Apr 2;19:96. doi: 10.1186/s12909-019-1529-7 (PMC6446288; doi:10.1186/s12909-019-1529-7)
Supplement: Supplementary file 2 — Focus-group interview formats translated into English. (DOCX 18 kb) [file 12909_2019_1529_MOESM2_ESM.docx]

Supplement 2. Focus-group interview formats translated into English

**Format for the focus-group interviews with the 1st year students in February 2014**

1. Studying

- What did you expect of studying when you started?
- How do you study independently?
- How do you study within your teaching group?
- How do you use iPad in PBL tutorials?
- How do you use iPad in lectures and practices?
- How do you use iPad on campus outside classes?
- How about outside the campus?
- When you study, do you use social media? Does it support and/or hinder your studies?
- How do you take notes? How do you organise your notes? How do you share your notes?
- How do you use learning materials, Inkling books and other learning materials?

2. Studying for the exams

- How do you retrieve?
- What is the most effective way of studying for the exams?
- How do you plan your use of time?
- How do you feel about studying for the exams?
- How well are the exams aligned with the content of your studies?
- How do you learn the key concepts?

3. New ideas for teaching and learning

- What wishes do you have for improving your studies?
- What ideas do you have for using the tablet computer in your studies?
- What ideas do you have for learning in class?
- What ideas do you have for improving the exams?
- How could you make use of social media in your studies?

**Format for the focus-group interviews with the 3^rd^ year students in May 2016**

**1. Mobile learning**

- What does mobile learning mean to you?
- What devices do you use in mobile learning?

**2. Digital learning environment**

- Describe the digital learning environment in the clinical studies, e.g. Moodle
- What is a good digital learning environment like?

**3. Pre-assignments**

- What kind of pre-assignments have you been offered (tests, videos, tasks etc.)?
- Have they been iPad compatible?
- What are good pre-assignments like?

**4. Learning materials on the Internet**

- Do the teachers upload the learning materials in the digital learning environment (Moodle or Dikki)?
- Are the learning materials uploaded in a Pdf format one slide per page?

**5. Studying**

- How do you use iPads in class?

**6. Teaching**

- How do the teachers use iPads in lectures?
- How do teachers use iPads in other type of teaching (group learning, skills labs etc.)?

**7. Exams and tests**

- How have you used iPads in exams and/or tests?

**8. Social media**

- How do you use social media to support your studies?
- What kind of social media groups do you have for studying?
- Do you use social media for groupwork?
- Would you like the teachers to establish Face book groups for studies?

**9. Teaching and learning with the patients**

- How do you use iPads in bedside/chairside teaching with patients?
- How do you take notes in bedside/chairside teaching with patients?
- Do you use another computer for information seeking?
- How could the use of iPads or other mobile devices be developed in bedside/chairside teaching with patients?

**10. Medical applications for clinical studies**

- Have the teachers recommended you good clinical applications?
- Have you discovered or your peers recommended you quality applications for clinical studies?
- Do you have ideas of how to use applications to support the patient’s self-management?

**11. Mobility**

- Where do you use iPads for studying during a normal day?
- How does the mobility of the device help you studying?

**12. Vision**

- What ideas do you have for the use of the mobile devices in the clinical practice of a physician/dentist?

**13. Message to the faculty**

- What message do you have to the faculty?
